# Finger-Actuated Microfluidic Concentration Gradient Generator Compatible with a Microplate

**DOI:** 10.3390/mi10030174

**Published:** 2019-03-02

**Authors:** Juhwan Park, Hyewon Roh, Je-Kyun Park

**Affiliations:** Department of Bio and Brain Engineering, Korea Advanced Institute of Science and Technology (KAIST), 291 Daehak-ro, Yuseong-gu, Daejeon 34141, Korea; juhwan3275@kaist.ac.kr (J.P.); hyewonr@kaist.ac.kr (H.R.)

**Keywords:** 96-well microplate, concentration gradient, enzyme kinetics, finger actuation, Hanes–Woolf plot, pneumatic valve, power-free microfluidics

## Abstract

The generation of concentration gradients is an essential part of a wide range of laboratory settings. However, the task usually requires tedious and repetitive steps and it is difficult to generate concentration gradients at once. Here, we present a microfluidic device that easily generates a concentration gradient by means of push-button actuated pumping units. The device is designed to generate six concentrations with a linear gradient between two different sample solutions. The microfluidic concentration gradient generator we report here does not require external pumps because changes in the pressure of the fluidic channel induced by finger actuation generate a constant volume of fluid, and the design of the generator is compatible with the commonly used 96-well microplate. Generation of a concentration gradient by the finger-actuated microfluidic device was consistent with that of the manual pipetting method. In addition, the amount of fluid dispensed from each outlet was constant when the button was pressed, and the volume of fluid increased linearly with respect to the number of pushing times. Coefficient of variation (CV) was between 0.796% and 13.539%, and the error was between 0.111% and 19.147%. The design of the microfluidic network, as well as the amount of fluid dispensed from each outlet at a single finger actuation, can be adjusted to the user’s demand. To prove the applicability of the concentration gradient generator, an enzyme assay was performed using alkaline phosphatase (ALP) and *para*-nitrophenyl phosphate (*p*NPP). We generated a linear concentration gradient of the *p*NPP substrate, and the enzyme kinetics of ALP was studied by examining the initial reaction rate between ALP and *p*NPP. Then, a Hanes–Woolf plot of the various concentration of ALP was drawn and the *V*_max_ and *K*_m_ value were calculated.

## 1. Introduction

Microfluidic technologies are widely used in clinical diagnostics and biomedical engineering fields. Small volumes of reagents can be rapidly and precisely controlled and reacted with each other, which makes the conventionally used analysis procedures efficient. Among the various microfluidic technologies, concentration gradient generation is one of the most useful technologies that can develop various concentration gradients at once, without the need for repetitive manual pipetting [1]. To date, microfluidic devices have been widely applied to generate the concentration gradient in various fields, such as cell-based drug screening [2,3], oil production screening from microalgae [4], immunohistochemistry [5], and antimicrobial susceptibility testing [6]. The different concentration of reagents is realized on a microscale, allowing rapid and high-throughput analysis compared to a bulk scale environment. By adjusting the geometry of microfluidic channels, many kinds of concentration gradient patterns can be achieved.

Although concentration gradient generation using a microfluidic device can be a powerful tool to enhance the research environment, there are two limitations that microfluidic concentration gradient generation have to overcome in order to be widely used. First, the expensive and complicated external pumping system is required for the flow generation of reagents [2,3,4,5,6]. For complicated microfluidic applications such as droplet generation [7] and inertial microfluidics [8], which require accurate control of the fluid, the external pumping system should be used. However, it is not appropriate to use the external pumping system for a simple flow operation which does not require flow rate control such as injection and drawing of fluid. To remove the needs of a complicated external pumping system, several simple pumping methods have been developed for microfluidic devices by applying capillary forces [9], vacuum driven pressure [10,11], and acoustic force [12]. In fact, generation of a pumpless microfluidic concentration gradient has also been reported by applying pipette-driven flow [13] and capillary flow on the paper substrate [14]. Second, several microfluidic devices for concentration gradient generation are limited to the laminar flow environment. Although concentration gradient generation in the laminar flow environment is beneficial to analyze the effects of various concentrations of reagents to the biological microenvironment, it is difficult to perform chemical and biological assays requiring the measurement of optical density due to the needs of specialized optical detection systems. In this context, the microfluidic chips should be compatible with conventional multiwell plates designed for conventional optical detection systems. Therefore, a few approaches have been proposed to develop microplates, including the microfluidic concentration gradient generator compatible with the microplate reader [15,16]. However, the area of the microfluidic channel reduces the number of wells in the microplate that can be analyzed, hindering high-throughput analysis, which is one of the advantages of microplates.

To overcome the above mentioned limitations, we report a microplate compatible microfluidic concentration gradient generator, which is simply actuated by finger motion. Previously, several approaches to simply operate microfluidic devices with finger motion have been proposed, but the operation of the device can be affected depending on the personal characteristics of finger motion [17,18,19]. To decrease the user-dependent variation, our group recently reported on the novel working principle of a finger-actuated microfluidic device [20]. The constant volume of fluid can be dispensed regardless of the various characteristics of finger actuation by the end-user. By applying this working principle, the finger-actuated concentration gradient generator was designed. The microfluidic networks are designed to generate six concentrations with a linear gradient between two reagents. A constant volume of reagents with a linear concentration gradient can be dispensed into the microplate. Finally, to verify this concept, we studied the enzyme kinetics in a 96-well microplate by simply generating a linear concentration gradient of the substrate.

## 2. Materials and Methods

### 2.1. Design and Concept

**Figure 1:**
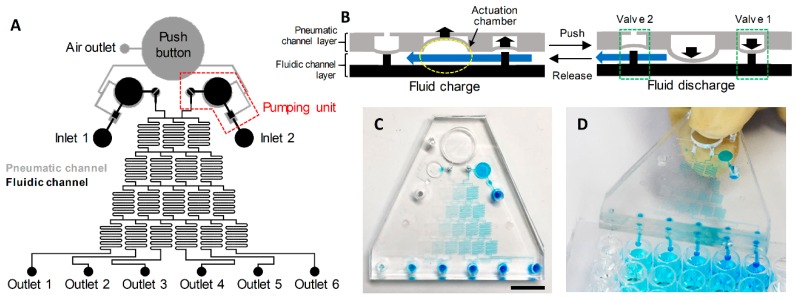
Design and working principle of the finger-actuated concentration gradient generator. (**A**) Drawing of the device design. (**B**) Operation principle of the pumping unit. The red dotted box in panel A indicates the single pumping unit. (**C**) Six concentrations with a linear gradient are generated from the device. Scale bar = 1 cm. (**D**) The picture of the device operation on the 96-well microplate.

The finger-actuated concentration gradient generator is designed to generate six concentrations with a linear gradient from two reagents (Figure 1A). Two reagents are simultaneously injected into the microfluidic networks to generate a linear concentration gradient by two finger-actuated pumping units operated by a single push button. The reagents from each inlet are mixed effectively by flowing through the mixing channels, before being dispensed from the outlet. Before operating the device, the air outlet is blocked with an acrylic block after pushing the push button to make a negative pressure in the pneumatic channel. As shown in Figure 1B, two pneumatic valves and an actuation chamber comprise the finger-actuated pumping unit and the operation of finger-actuated pumping unit is based on the deflection of the thin poly(dimethylsiloxane) (PDMS) membrane. The PDMS membrane at the pneumatic valve 1 and the actuation chamber are actuated by the pressure change at the pneumatic channel. However, since the pneumatic valve 2 is not connected to the push button by the pneumatic channel, it is actuated according to the pressure change at the fluidic channel. When the button is pushed, increased pressure in a pneumatic channel deflects the PDMS membrane that closes the valve 1 and compresses the actuation chamber. Then, the fluid in an actuation chamber is dispensed into the outlet by opening the pneumatic valve 2. Conversely, the fluid is charged into the actuation chamber when the button is released. The pneumatic valve 2 is closed due to the decreased pressure in a fluidic channel since the pneumatic valve 1 is opened and the actuation chamber is decompressed. Consequently, the fluid can be repeatedly charged and discharged without backflow by pushing and releasing the button. After loading the reagents into each inlet, the device is operated by pushing the button as needed by the end-user. Two fluids are simultaneously injected into the microfluidic networks and six fluids that have different concentration gradients are dispensed into the outlet. The amount of fluid loaded into the inlet can be modified to the needs of the end users, depending on the desired total volume dispensed from the outlet, but the volume dispensed after every single actuation is consistent. However, since the actuation chambers meter the accurate amount of fluid injected into the microfluidic networks, no micropipette is required to inject the correct amount of fluid into the inlet. The volume of the actuation chamber is designed to be 3 μL, so that each time the device is actuated, 1 μL of fluid is dispensed into each outlet. The position of each outlet is designed to be compatible with 96-well microplate and the Tygon tubes are connected to the outlet to facilitate the reagents loading into the microplate. As shown in Figure 1C,D, the desirable volume of fluids having a linear concentration gradient can be simultaneously loaded into the 96-well microplate.

### 2.2. Device Fabrication

**Figure 2:**
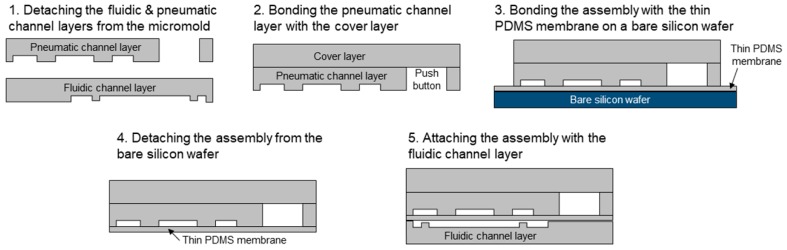
Fabrication processes of a finger-actuated microfluidic device.

The finger-actuated microfluidic concentration gradient generator consists of three PDMS layers (thickness = 3 mm) and one thin PDMS membrane layer (thickness = 25 μm) (Figure 2). The three PDMS layers were obtained by pouring a mixture of PDMS precursors and a curing agent (PDMS:curing agent = 10:1) onto an SU-8 mold fabricated through photolithography. The PDMS mixture was baked at 85 °C for 60 min and then peeled off. The SU-8 mold was obtained by two-step lithography to make a gap between the weir structure of pneumatic valves and the thin PDMS membrane, which can prevent the weir structure of pneumatic valve from bonding to the thin PDMS membrane. First, SU-8 2005 (MicroChem Corp., MA, USA) was spin-coated onto a silicon wafer (thickness = 5 μm), and then SU-8 2100 was spin-coated (thickness = 95 μm) to obtain a thickness of 100 μm. The thin PDMS membrane layer was obtained by spin-coating the mixture of PDMS onto a bare silicon wafer at a speed of 1500 rpm for 60 s and then baked at 150 °C for 10 min. The bonding of the layers and the membrane was attained through oxygen plasma treatment of 1 min and incubation in an oven at 65 °C for 10 min. The top two PDMS layers were first assembled, then the joined layers were bonded with the PDMS membrane attached to the bare silicon wafer. After peeling off the assembly from the bare silicon wafer, it was then finally bonded with the bottom PDMS layer through oxygen plasma treatment. To fabricate the device to be compatible with a commonly used 96-well microplate, a thin Tygon tube was cut into 6 pieces (length = 1.5 cm) and each inserted into the outlet of the device for the dispensed solution to reach into the well.

### 2.3. Analysis of the Concentration and Volume of the Sample Solution

To analyze the concentration of the dispensed sample solution, 10 μL of 0 to 1 mM erioglaucine (Sigma-Aldrich, St. Louis, MO, USA) with an interval of 0.1 mM, which has a peak absorbance at 406 nm, were manually pipetted into the 96-well plate containing 200 μL of distilled water. The absorbance of the mixture was measured at 406 nm with a microplate reader (SpectraMax 250; Molecular Devices, Sunnyvale, CA, USA), then the calibration curve with respect to the concentration of erioglaucine was attained. The two inlets of the microfluidic device were each injected with distilled water and 1 mM erioglaucine solution. With the finger actuation of the device, 10 μL of dispensed sample solution was ejected from each of the six outlets. Then, the dispensed sample solution was loaded into the 200 μL of distilled water located in the well of a conventional 96-well microplate. The absorbance of the mixture was measured at 406 nm, then the concentration of the mixture was calculated using the calibration curve.

To analyze the volume of the dispensed solution, 1 to 20 μL of 1 mM of erioglaucine with an interval of 1 μL were manually pipetted into the 96-well plate containing 200 μL of distilled water. By measuring the absorbance of the mixture at 406 nm, the calibration curve with respect to the volume of 1 mM erioglaucine was obtained. The absorbance of the dispensed solution from all six outlets through one to four times of finger-actuation of the device was measured after mixing the dispensed solution with 200 μL of distilled water. Then, the volume of the dispensed solution of the device was calculated using the obtained calibration curve.

### 2.4. Enzyme Assay

To demonstrate the applicability of the microfluidic device, enzyme assay was conducted with alkaline phosphatase (ALP) (Sigma-Aldrich) and *para*-nitrophenyl phosphate (*p*NPP) (Sigma-Aldrich). We prepared 53.89 mM of *p*NPP substrate solution using a glycine buffer (0.1 M, pH = 10.4). Then, 100, 150, 200 U/L of ALP enzyme solution were prepared using a 1 mM magnesium chloride buffer solution. To generate a linear concentration gradient of *p*NPP, glycine buffer solution and 53.89 mM of *p*NPP was each injected into two inlets of the microfluidic device. Then, the device was operated to dispense 2 μL of *p*NPP from each six outlets, which were then transferred to 60 μL of ALP in a 96-well microplate. The absorbance of the mixture was measured every 15 s over a period of 1 min using the microplate reader, and the shaking of the well plate was also performed between the measurements to improve the mixing of the ALP and the diluted *p*NPP. Since the *p*NPP is decomposed by ALP to produce 4-nitrophenol and phosphoric acid with the same reaction ratio, the decomposed amount of *p*NPP is same as the amount of generated *p*-nitrophenol. Hence, the initial reaction rate between ALP and *p*NPP was measured through obtaining the slope of the curve drawn by converting the measured absorbance to the generated concentration of *p*-nitrophenol using a calibration curve. The above procedure was conducted by varying the enzyme concentration from 100 to 200 U/L. The measured initial reaction rate was then analyzed for enzyme kinetics study using the Hanes–Woolf plot for various concentrations of ALP.

## 3. Results and Discussion

### 3.1. Assessment of the Device Operation

To verify the generation of the linear concentration gradient in the device, we compared the optical density of fluids at each outlet with the concentration gradient generated by manual pipetting. Distilled water was injected into the inlet 1 and the 1 mM erioglaucine solution was injected into the inlet 2. Theoretically, the concentration of mixture at each branch point of the microfluidic network can be calculated as the following equation [15].

(1) $$C\left(i,N\right)=\frac{\left(N-i\right)C_{1}+iC_{2}}{N}$$

where *C(i,N)* is the concentration at the end of the serpentine microfluidic channel, *N* means the number of the column in the microfluidic network (*N* = 1, 2, 3, 4, 5), *i* means the number of the row in the microfluidic network (*i* = 0, 1, 2, 3, 4, 5). *C*_1_ and *C*_2_ mean the initial concentration of the reagent from each inlet.

**Figure 3:**
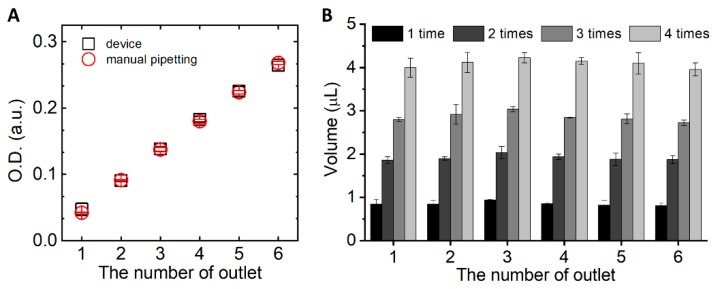
Estimation of concentration gradient generation. (**A**) The optical density of the dispensed solutions from each outlet was compared between the manual pipetting method and the finger-actuated concentration gradient generator. The error bars represent the standard deviation of three replicates. (**B**) The dispensed volume from each outlet was compared with respect to the number of the pushing times. The error bars represent the standard deviation of three replicates.

Since the 1 mM erioglaucine solution was injected into the inlet 2 and the distilled water was injected into the inlet 1, it is expected that 0, 0.2, 0.4, 0.6, 0.8, and 1 mM of erioglaucine solution is achieved from the outlet 1, 2, 3, 4, 5, and 6, respectively. Therefore, the optical density of manually prepared sample solution was compared to the sample solution prepared by the finger-actuated concentration gradient generator (Figure 3A). The optical density of both cases represented by concentration increased linearly and there was no significant difference between the two methods. Ideally, only distilled water that was injected into the inlet 1 should be dispensed from the outlet 1. However, there was a little increase in optical density from the outlet 1 which means a little amount of erioglaucine was dispensed into the outlet 1. It is supposed that there was a little time difference between the compression of each actuation chamber although the actuation chambers are designed to be actuated at the same time. In addition, the spiking of erioglaucine solution into the outlet 1 can be caused by the diffusion of erioglaucine solution to the actuation chambers of distilled water during the interval between the pushing of the button.

The device can generate various concentrations with a linear gradient faster than manual pipetting when the end users require a small amount of reagents. The device can dispense 1 μL of reagents to each outlet at once and it takes about 2 s to hold the button. Before dispensing the different concentrations of reagents to the microplate, the device should be actuated about seven times to fill the microfluidic channels with reagents, and it takes about 15 s. The time to generate a concentration gradient on the device would increase as the desired volume increases, while the time does not increase when the number of concentrations increases. On the other hand, it takes about 130 s to generate six concentrations with a linear gradient by manual pipetting. The time to generate a concentration gradient by manual pipetting would not increase as the desired volume increases, but the time increases as the number of concentration increases. Therefore, concentration gradient generation on the device is beneficial when the end users need a small volume of reagents with a large number of concentrations.

The device introduced here can generate six concentrations, but the design of the device can be modified to have a different number and position of the outlets to meet the needs of end users. If the fluidic resistance increases due to the increased number of concentrations, the time required to fully compress the actuation chambers will be longer. Therefore, end users may have to hold the button for a longer period of time. The minimum holding time of the button would be a little bit different according to the design of the microfluidic channels. In this manner, the instruction regarding the minimum holding time of the button should be provided according to the design of microfluidic networks.

On the other hand, the adjustment of dispensable volume is not possible without re-fabrication of the micromolds. The dispensable volume is determined by the squeezed volume of the actuation chamber which is determined by the squeezed volume of the pressure chamber. The minimum volume of the squeezed pressure chamber with a human finger actuation is 41 μL [20]. Therefore, the volume of the actuation chambers should be designed less than 41 μL to dispense a constant volume regardless of the personal characteristics of the finger actuation. The novelty of our system is to reduce the user-dependent variation due to the personal characteristics of the finger actuation by limiting the squeezed amount of the actuation chamber. The height of pneumatic and fluidic channels plays a role as a limit to the compressed and decompressed amount of the actuation chamber. The squeezed volume of the actuation chamber can be controlled if less than 41 μL of the pressure chamber is squeezed by finger actuation, but it is impossible. Therefore, the micromolds should be re-fabricated to adjust the amount of the dispensed volume.

Additionally, it was assessed whether uniform amount of the reagent is dispensed into each outlet or not. To ensure that the same amount of fluid would come out at each outlet, the geometry of microfluidic channels connected to each outlet was designed to have the same fluidic resistance. As shown in Figure 3B, a constant amount of fluid was dispensed into each outlet when the button was pressed and the volume of fluid increased linearly as the number of device actuation increased. The dispensed volume of reagents can be simply controlled according to the number of actuation. Actually, the device was designed to dispense 1 μL of fluid into each outlet once the button is pressed. However, as shown in Table 1, there were errors in comparison with the target value. It is supposed that the errors are induced by the difference between the targeted and actual height of micromold. Although we targeted the height of micromold to achieve a 3 μL volume of the actuation chamber, it was difficult to fabricate the height of micromold exactly the same as the targeted value. We expect the errors due to the fabrication procedures to be improved when the fabrication method would be changed to more exquisite methods such as injection molding or 3D printing. Additionally, for one-time pushing, the error and CV were quite larger than those of more pushing times of the button. The volume of dispensed reagents from the device was analyzed by transferring fluids to the microplate using the micropipette. We suppose that the inaccuracy is larger when handling with a low volume of fluid due to the errors arising during the process of fluid acquisition by micropipette. 

### 3.2. Enzyme Kinetics Study

**Figure 4:**
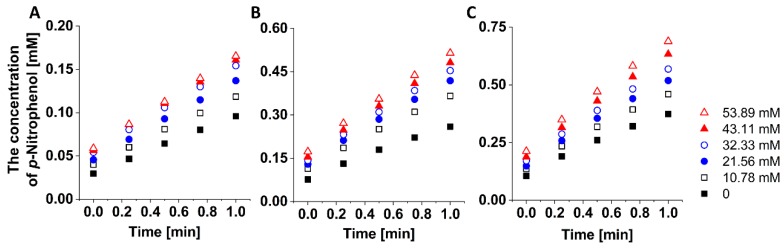
The concentration of *p*-nitrophenol was measured for 1 min to determine the initial reaction rate for the various enzyme concentrations. (**A**) 100 U/L. (**B**) 150 U/L. (**C**) 200 U/L.

**Figure 5:**
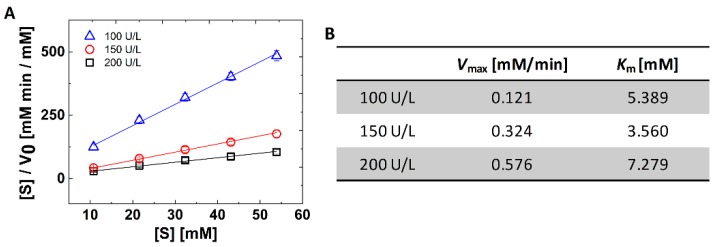
The results of the enzyme kinetics study. (**A**) The Hanes–Woolf plot for the various concentrations of ALP. The error bars represent the standard deviation of three replicates of the assay. (**B**) *V*_max_ and *K*_m_ value of each enzyme unit were obtained from the linear fitting of panel A.

To study the enzyme kinetics of ALP, 53.89 mM of *p*NPP was injected into the inlet 1 and the buffer solution was injected into the inlet 2. Since the microfluidic network was designed to generate a linear concentration gradient, it is expected that the 0, 10.78, 21.56, 32.33, 43.11, 53.89 mM of *p*NPP can be achieved in each outlet. After generating the linear concentration gradient of *p*NPP, the enzyme kinetics of ALP was investigated by measuring the initial reaction rate between ALP and *p*NPP. Since the *p*-nitrophenol and phosphoric acids were generated from *p*NPP under ALP, the reaction rate was calculated by the generation rate of *p*-nitrophenol. As shown in Figure 4, the concentration of *p*-nitrophenol increased linearly with respect to the reaction time and the concentration of *p*-nitrophenol increased with respect to the concentration of ALP. Although the microfluidic networks were designed to generate 0 mM of *p*NPP in the outlet 1, a small amount of *p*NPP was injected into the outlet 1 due to the time difference between the compression of two actuation chambers as we above mentioned. Therefore, we calculated the initial reaction rate at the outlet 2, 3, 4, 5, and 6 for 1 min. From the calculated initial reaction rate, a Hanes–Woolf plot for the various concentrations of ALP was achieved with the following equation (Figure 5A).

(2) $$\frac{\left[S\right]}{v_{0}}=\left(\frac{1}{V_{\max}}\right)\left[S\right]+\;\frac{K_{m}}{V_{\max}}$$

where [*S*] is the concentration of *p*NPP, *v*_0_ is the initial reaction rate, *V*_max_ is the maximum velocity of ALP, and *K*_m_ is the Michaelis constant. In the Hanes–Woolf plot, the reciprocal of slope means *V*_max_ and the intercept with *y* axis means *K*_m_ over *V*_max_. Therefore, we calculated the *V*_max_ and *K*_m_ value with respect to the concentration of ALP (Figure 5B). The calculated *K*_m_ value shows the agreement with the other studies under the same order [21,22].

Theoretically, the *V*_max_ increases linearly according to the concentration of enzyme while the *K*_m_ value shows a constant value regardless of the concentration of the enzyme. However, as shown in Figure 5B, the *K*_m_ value does not show a constant value while the *V*_max_ increases linearly. It can be supposed that only 2 μL of the *p*NPP from each outlet was used to measure the initial reaction rate. Since the finger-actuated concentration gradient generator is not elaborate compared to the complicated external pumping system, errors may occur when the number of actuation is small. Actually, when verifying the operation of the device using the 1 mM erioglaucine solution, there were no significant errors because a quite large volume (10 μL) of the reagent from each outlet was analyzed from each outlet (Figure 3A). We expect that the errors regarding *K*_m_ value can be corrected by using larger amounts of reagents.

## 4. Conclusions

In this work, a finger-actuated linear concentration gradient generator was described. The distance between each outlet was designed to be the same as the distance between each well of the 96-well plate, which enables the direct transfer of diluted solutions to the microplate wells. Only finger motion can actuate the flow in microfluidic channels without the use of any external equipment. The linear concentration gradient between two reagents was successfully generated and constant volume of reagents was dispensed from each outlet. The concentration gradient pattern and the design of microfluidic network can be adjusted depending on the need of the end-users. Additionally, the amount of fluid dispensed at a single finger actuation can also be adjusted. Furthermore, the number of outlets as well as the location is controllable according to the end-users’ need. As a proof of the concept, the finger-actuated linear concentration gradient generator was used to study the enzyme kinetics of ALP. The linear concentration gradient of the substrate was easily generated by just using the finger motion without manual pipetting. The results of enzyme assay could be measured by conventional microplate reader without the fabrication of special optical detection systems. We expect the finger-actuated concentration gradient generator to be a convenient tool for performing various chemical and biological assays in the conventional laboratory.

## Figures and Tables

**Table 1:** Statistical analysis regarding the dispensed volume from each outlet with respect to the number of the pushing times.

	One-Time Pushing (1 μL)				Two-Time Pushing (2 μL)				Three-Time Pushing (3 μL)				Four-Time Pushing (4 μL)			
	Mean (μL)	Standard Deviation	CV (%)	Error (%)	Mean (μL)	Standard Deviation	CV (%)	Error (%)	Mean (μL)	Standard Deviation	CV (%)	Error (%)	Mean (μL)	Standard Deviation	CV (%)	Error (%)
**Outlet 1**	0.847	0.103	12.206	15.278	1.861	0.071	3.854	6.9195	2.797	0.048	1.742	6.762	3.995	0.215	5.403	0.111
**Outlet 2**	0.846	0.080	9.501	15.327	1.896	0.036	1.912	5.1585	2.918	0.224	7.683	2.711	4.117	0.230	5.597	2.926
**Outlet 3**	0.939	0.007	0.796	6.002	2.032	0.140	6.915	1.637	3.038	0.058	1.938	1.289	4.226	0.119	2.816	5.654
**Outlet 4**	0.853	0.021	2.552	14.633	1.939	0.0659	3.396	3.001	2.845	0.006	0.239	5.158	4.147	0.078	1.884	3.695
**Outlet 5**	0.818	0.110	13.539	18.105	1.879	0.146	7.792	6.002	2.813	0.114	4.057	6.233	4.096	0.248	6.071	2.418
**Outlet 6**	0.808	0.065	8.115	19.147	1.872	0.091	4.871	6.399	2.726	0.061	2.256	9.110	3.957	0.147	3.726	1.054

* Error (%) = $\frac{\left|\mathrm{Target}\;\mathrm{value}\;–\;\mathrm{Mean}\;\mathrm{value}\right|}{\mathrm{Target}\;\mathrm{value}}\times 100$.
